# The MK2/HuR signaling pathway regulates TNF-α-induced ICAM-1 expression by promoting the stabilization of ICAM-1 mRNA

**DOI:** 10.1186/s12890-016-0247-8

**Published:** 2016-05-23

**Authors:** Ting Wu, Jia-Xin Shi, Shen Geng, Wei Zhou, Yi Shi, Xin Su

**Affiliations:** Department of Respiratory and Critical Care Medicine, Jinling Hospital, Nanjing University School of Medicine, 305 East Zhongshan Road, Nanjing, 210002 China; Department of Respiratory Medicine, Lianyungang First People’s Hospital, Affiliated Hospital of Xuzhou Medical College, Clinical Medical School of Nanjing Medical University, Lianyungang, 222002 China; Department of Respiratory and Critical Care Medicine, Jinling Hospital, Southern Medical University, Guangdong, 510000 China

**Keywords:** MK2, HuR, ICAM-1, IL-8, HPMEC

## Abstract

**Background:**

Acute lung injury (ALI) and acute respiratory distress syndrome (ARDS) are characterized by acute lung inflammation. Intercellular adhesion molecule-1 (ICAM-1) and interleukin-8 (IL-8) play an important role in the development of these diseases. Mitogen-activated protein kinase (MAPK) p38/activated protein kinase 2 (MK2) regulates the expression of ICAM-1 and IL-8 in human lung microvascular endothelial cells (HPMECs) stimulated by tumor necrosis factor-α (TNF-α); however, the underlying molecular mechanism remains unclear. Here, we show that human antigen R (HuR), an RNA binding protein which binds preferentially to AU-rich elements (AREs) and stabilizes mRNAs, regulates TNF-α-induced ICAM-1 expression in the MK2/HuR signaling pathway.

**Method:**

MK2 and HuR were silenced respectively in HPMECs and then HPMECs were stimulatied with TNF-α. Nucleo-cytoplasmic shuttling of HuR was detected by subcellular fractionation and confocal microscopy in MK2 knockdown HPMECs. In HuR silencing cells, protein and mRNA levels of ICAM-1 and IL-8 were measured by western blot analysis, ELISA and real-time PCR; mRNA stabilization were measured by real-time PCR after actinomycin D (ActD) blocking transcription. Furthermore, we performed neutrophil adhesion assay to assess the adhering capacity after HuR silencing.

**Results:**

MK2 were subjected to a knockdown by interfering RNA, the mRNA and protein levels of HuR in human pulmonary microvascular endothelial cells (HPMECs) were not affected. However, after the stimulation of TNF-α, silencing MK2 inhibited HuR accumulation to cytoplasm from nucleus in HPMECs. Consequently, knockdown of HuR by RNA interference in HPMECs, there was reduction in the stability of ICAM-1 mRNA and ICAM-1 protein level. This event was accompanied by a decrease in the adhesion of neutrophils towards HPMECs. Nevertheless, HuR silencing had no effect on the mRNA and protein levels of IL-8.

**Conclusion:**

These results indicate that MK2 post-transcriptionally regulates TNF-α-induced ICAM-1 expression by altering the cytoplasmic localization of HuR in HPMECs.

## Background

Acute lung injury (ALI) and acute respiratory distress syndrome (ARDS) are two acute inflammatory diseases. In the lungs, neutrophil recruitment and activation play a pivotal role in ALI/ARDS [1, 2]. Tumor necrosis factor-α (TNF-α), an important proinflammatory factor of ALI/ARDS [3], stimulates the expression of intercellular adhesion molecule-1 (ICAM-1) and interleukin-8 (IL-8) in human pulmonary microvascular endothelial cells (HPMECs) [4–6]. Both ICAM-1 and IL-8 are the most important inflammatory mediators that are responsible for the migration, recruitment, and infiltration of neutrophil that are produced to combat acute lung inflammation in humans [7, 8]. Our previous study showed that mitogen-activated protein kinase (MAPK) p38/ MAPK-activated protein kinase 2 (MK2) regulates the expression of ICAM-1 and IL-8 at the post-transcriptional level in HPMECs after TNF-α stimulation [4]. However, the precise mechanism by which MK2 regulates ICAM-1 and IL-8 expression remains elusive.

Previous studies have shown that the stability of mRNA containing AU-rich elements (ARE)(AU is the abbreviated form of adenylate uridylate) is regulated through an MK2-dependent and ARE-targeted mechanism [9–11]. Moreover, MK2 plays an important role in regulating the expression of TNF-α and IL-6 [11–13]. However, the downstream proteins by which MK2 stabilizes ARE-containing mRNAs are largely unknown. HuR, a member of the embryonic lethal abnormal visual family, can bind to the ARE motif, such as AUUUA in the 3′-UTR of mRNA [14–17]. Recent studies have shown that HuR increases the mRNA stability of survival motor neuron (SMN) protein and toll-like receptor 4 (TLR4), which are located in motor neuron derived (MN-1) cells and vascular smooth muscle cells (VSMCs), respectively [18, 19]. Furthermore, it has been reported that active MK2 induces cytoplasmic accumulation of HuR, which correlates with increased stability of urokinase plasminogen activator (uPA) and uPA receptor mRNAs that have AREs in their 3′-UTR [20, 21]. Considering that AREs located in the 3′-UTR of ICAM-1 and IL-8 mRNAs, we hypothesize that the MK2 pathway might regulate the expression of ICAM-1 and IL-8 through HuR in TNF-α-induced HPMECs. In this study, we have investigated the intracellular localization of HuR following silencing of MK2 and determined ICAM-1 and IL-8 the expression after knockdown of HuR in TNF-α stimulated HPMECs. We found that TNF-α stimulation induced cytoplasmic accumulation of HuR, which was significantly suppressed by MK2 silencing. Importantly, knockdown of HuR destabilized ICAM-1 mRNA and reduced ICAM-1 protein levels. Our study revealed that MK2 mediates expression of ICAM-1 with TNF-α stimulation by increasing the cytoplasmic accumulation of HuR in HPMECs.

## Methods

### Materials

Actinomycin D (ActD) was purchased from Sigma-Aldrich (St. Louis, USA). Lipofectamine 2000 (11668–027) and Opti-MEM I reduced serum medium were obtained from Invitrogen (Carlsbad, USA). MK2 siRNA, HuR siRNA and negative control siRNA were received from GenePharma (Shanghai, China). TNF-α (300-01A) was purchased from PeproTech (Rocky Hill, USA). Phosphatase Inhibitor Cocktail (04906845001) was from Roche (Mannheim, Germany). An enhanced chemiluminescent (ECL) kit were obtained from Pierce (Rockford, USA). The protein assay kit, nuclear and cytoplasmic protein extraction kit (P0027) and the RIPA Lysis Buffer (P0013C) were purchased from Beyotime Institute of Biotechnology (Nantong, China). Antibodies raised against MK2 (#3042), phospho-MK2 (Thr334, #3041), ICAM-1 (#4915) was obtained from Cell Signaling (Danvers, USA); a monoclonal antibody against HuR (ab14371) was received from Abcam (Cambridge, USA); antibodies against β-actin (P30002) and histone (P30266) were from Abmart (Shanghai, China);IL-8 ELISA Kit (BMS204/3) was received from eBioscience (San Diego, USA). RevertAid First Strand cDNA Synthesis Kit (#K1622) was purchased from Fermentas UAB (Vilnius, Lithuania). FastStart Universal SYBR Green Master (Rox) (04913914001) was purchased from Roche (Mannheim, Germany). Endothelial cell medium and HPMECs were purchased from ScienCell (Carlsbad, USA).

### Cell culture, treatment, and transfection

HPMECs were grown in ECM medium supplemented with 5 % fetal bovine serum.MK2 siRNA and HuR siRNA were respectively reverse transfected at a concentration of 40 nm with Lipofectamine 2000 diluted in Opti-MEM. After 24 h, to allow for gene silencing, cells were stimulated with TNF-α. The final concentration of TNF-αwas 10 ng/ml. The sequences of siRNAs are as follows: MK2 siRNA 1: sense (5’-GAC CAG GCAUUC ACA GAA ATT-3’) and antisense (5’-UUU CUG UGA AUG CCU GGU CTT-3’); MK2 siRNA 2: sense (5’-CAC CCU UGG AUC AUG CAA UTT-3’) and antisense (5’-AUU GCA UGA UCC AAG GGU GTT-3’); MK2 siRNA 3: sense (5’- GUU AUA CAC CGU ACU AUG UUU-3’) and antisense (5’-ACA UAG UAC GGU GUA UAA CUU-3’); HuR siRNA: sense (5’-GGA UGA GUU ACG AAG CCU GTT-3’) and antisense (5’-CAG GCU UCG UAA CUC AUC CTG-3’). Controls were treated with negative control siRNA anLipofectamine 2000.

### Western blot

The total cellular lysates were prepared as previously described [22]. Cells were lysed with RIPA lysis buffer containing proteinase inhibitor. Cell lysates were mixed with loading buffer and boiled for 10 min, resolved by 10 % sodium dodecyl sulfatepolyacrylamide gel electrophoresis, and transferred onto a polyvinylidene fluoride membrane (PVDF). The membranes were blocked with 5 % bovine serum albumin (BSA). Western blot analysis was then performed in accordance with standard protocols using antibodies against MK2,HuR,ICAM-1, β-actin and histone, followed by incubation with relevant horseradish peroxidaseconjugated (HRP) secondary antibodies. The immune complexes were visualized on FluorChem FC2 system (Cell Biosciences, Santa Clara, USA). Densitometric analysis of bands was performed by ImageJ software (http://rsb.info.nih.gov/ij/, National Institutes of Health, USA), and the data were normalized to β-actin or histone.

### Subcellular fractionation

The Nuclear and Cytoplasmic Protein Extraction Kit (P0027) were purchased from Beyotime Institute of Biotechnology (Nantong, China). Nuclear and cytoplasmic protein fractions of HPMEC were obtained according to manufacturer’s directions. In brief, extraction reagent A for cytoplasmic protein was added with 1 mM phenylmethylsulfonyl fluoride (PMSF) and incubation on ice for 15 min. Then extraction reagent B for cytoplasmic protein was added. The cytosolic fractions were prepared by high-speed centrifugation (16000 g for 5 min at 4 °C) and the supernatant was collected. For preparing nuclear fractions, extraction reagent for nuclear protein with PMSF in supplemented was added to nuclear sediment, centrifuged (10 min, 16000 g, 4 °C) after 30 min, and the supernatant was saved.

### Confocal microscopy

HPMECs seeded on coverslips were transfected with siRNA or negative control siRNA as described above. After treatment with TNF-α, cells were washed using with phosphate buffered saline (PBS) and fixed with 4 % paraformaldehyde for 15 min at room temperature. The fixed HPMECs were permeabilized with 0.5 % Triton X-100 in PBS for 10 min at room temperature. Subsequently, they were blocked with 1 % BSA in PBST for 30 min at room temperature. Finally, they were washed with PBS and incubated overnight with primary HuR antibody (100-fold diluted with 1 % BSA in PBST) at 4 °C, followed by 3 × 5 min washes with PBS. The HPMECs were then incubated with fluorescein isothiocyanate (FITC)-labeled goat anti-mouse IgG (green) (200-fold diluted with 1 % BSA in PBST) for 1 h in the dark. Following 3 × 5 min washes with PBS, the 4’-6-diamidino-2-phenylindole (DAPI) was used to stain the cell nuclei (blue) for 5 min at a concentration of 1 μg/ml. After 3 × 5 min washes with PBS, the confocal images were acquired using an Olympus FV1000 confocal microscope (Olympus, Tokyo, Japan). FV10-ASW software (Olympus Europa, Japan) was used to process the images.

### Enzyme-linked immunosorbent assay (ELISA)

Cell-free supernatants were saved and aliquots were stored at −70 °C until use. The protein levels of IL-8 in medium were measured with commercially available cytokine specific ELISA kits (eBioscience, USA) according to the manufacturers’ recommendations.

### RNA isolation and Quantitative RT-PCR

Total RNA was isolated from cultured cells by TRIzol and used in reverse transcription and PCR amplification reactions as described [22]. Quantitative RT-PCR analysis was performed on an Applied Biosystems model 7300 real-time PCR machine. (Applied Biosystem, Foster City, USA). Data were analyzed by delta-delta Ct method and normalized to the housekeeping gene GAPDH. The primers used for the amplifications are as follows: GAPDH forward, TGCACCACCAACTGCTTAGC; GAPDH reverse, GGCATGGACTGTGGTCATGAG; HuR forward, CCGTCACCAATGTGAAAGTG; HuR reverse, TCGCGGCTTCTTCATAGTTT; ICAM-1 forward, AGCTTCTCCTGC TCTGCAAC; ICAM-1 reverse, GTCTGCTGGGAATTTTCTGG; IL-8 forward, ATG ACTTCCAAGCTGGCCGTGGCT; IL-8 reverse, TCTCAGCCCTCTTCAAAAACT TCTC.

### Analysis of mRNA stability by real-time PCR

50 % confluent cells were first preincubated with Actinomycin D (5 μg/ml) for 2 h; cells were then treated with TNF-α (10 ng/ml) for 2 h in the presence of actinomycin D. Total RNA was prepared as described [22]. HuR, ICAM-1 and IL-8 mRNAs levels were normalized to GAPDH mRNA. The normalized value at TNF-α 0 h was set as 100 %. GraphPad Prism software version 5.01 (GraphPad Software,Inc.,USA) was used to calculated the half-lives of mRNAs on a one-phase exponential decay model. The semi-logarithmic curves were also analysis by GraphPad Prism software.

### Neutrophil preparation

Neutrophil polymorphonuclear granulocytes (PMN) were isolated from the peripheral blood of healthy human volunteers from Lianyungang First People’s Hospital using Ficoll-Hypaque (Sigma, poole, UK) density gradient centrifugation, as previously described [23]. Residual erythrocytes were subjected to hypotonic lysis. The remaining neutrophils were washed and resuspended again in PBS such that their concentration in the suspension was 5x10^7^cells/ml. We used this neutrophilic suspension in PBS within 4 h.

### Neutrophil adhesion assay

PMN were labeled for 1 h at 37 °C with 10 mM 2',7'-bis-(2-carboxyethyl)-5-(and-6)-carboxyfluorescein,acetoxymethyl ester BCECF/AM (Beyotime, Haimen, China). HPMECs were grown in 12-well plates and transfected with siRNA or negative control siRNA as described above. After treatment with TNF-α to HPMEC, the labeled PMN were added to each HPMEC containing well and incubated for 1 h. Non-adherent PMN were removed by two gentle washes with PBS; The adhesion PMN cells were counted in five high-power fields per well under fluorescence microscope (Olympus BX61, Japan) and expressed as adhesion PMN per high-power fields.

### Statistical analysis

Data were expressed as either a representative experiment or the mean ± standard error (SE) of three independent experiments. Statistical analysis to determine significance was calculated using variance (ANOVA) and an unpaired *t* test two tailed, analyzing the differences between the different time points within the same treatment group and between different groups, respectively. *P* values < 0.05 was consideredstatistically significant. Statistical analysis was performed by SPSS 13.0 (IBM SPSS, Chicago, USA).

## Results

### Knockdown of MK2 by siRNA had proved to be inoperative on HuR expression

**Fig. 1:**
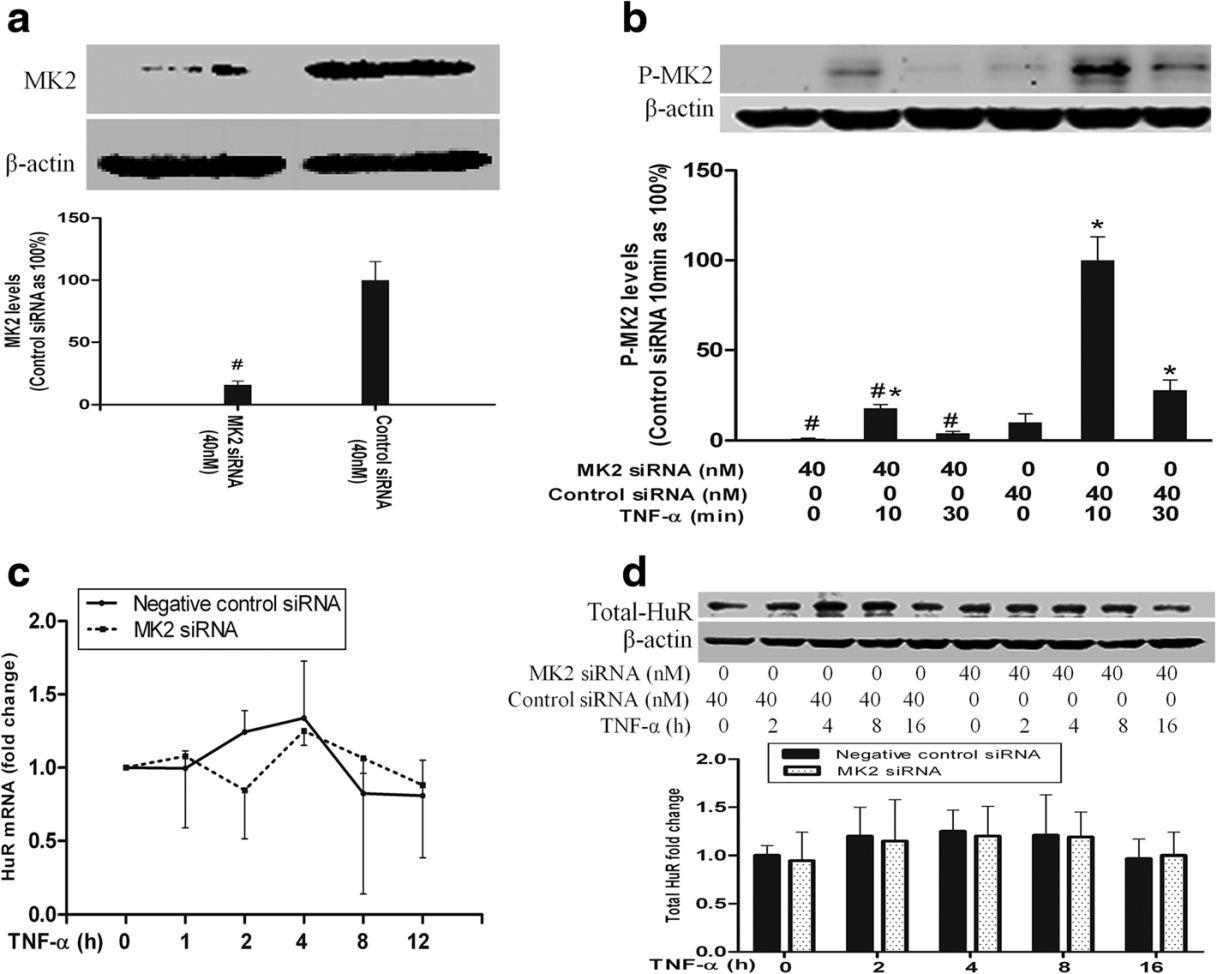
The effect of MK2 on TNF-α-induced HuR expression in HPMECs. The Western blot band density data showed that MK2 knockdown greatly reduced the levels of MK2 (**a**) and phospho-MK2 (**b**). MK2 silencing had no effect on HuR mRNA levels (**c**) and HuR protein levels (**d**). Band density and mRNA data are expressed as mean ± SEM. n =3, #*P* <0.05 vs. negative control siRNA-transfected cells; *P <0.05 vs. cells at TNF-α 0 h. Control siRNA, negative control siRNA; P-MK2, phospho-MK2

It was reported that activation of p38 MAPK *via* IL-1β and TNF-α is implicated in HuR-mediated stabilization of mRNAs coding for key inflammatory mediators including IL-6, cyclooxygenase-2 (COX-2) and TNF-α [24]. MK2 has been shown crucial for HuR translocation [24, 25], but it remains unknown whether MK2 affects the expression of HuR mRNA and protein after TNF-α activation. To assess the role of MK2 in HuR expression, we applied MK2 siRNA (40 nM) to silence MK2, and then the HPMECs were stimulated with TNF-α (10 ng/ml). After 0, 1, 2, 4, 8 and 12 h, RNA and protein were extracted for real-time PCR and immunoblotting. The Western immunoblot analysis manifest that MK2 knockdown remarkably weakened MK2 expression level to ~18 % of controls (Fig. 1a). Phosphorylation of MK2 is essential for MK2 activtion. MK2 siRNA reduced the phosphorylation MK2 under normal growth condition (Fig. 1b). TNF-α stimulation for 10 and 30 min obviously increased the phosphorylation of MK2, which was apparently reduced by MK2 siRNA (Fig. 1b). Next, we assessed HuR mRNA expression in HPMECs after TNF-α stimulation in condition of MK2 knockdown. As shown in Fig. 1c, HuR mRNA levels did not change significantly after TNF-α stimulation both in control groups and in MK2-silencing groups, a difference that was not statistically significant. To test whether MK2 influences HuR protein levels, total cellular proteins were prepared for immunoblotting of HuR. Silencing of MK2 did not influence HuR protein levels in the presence or absence of TNF-α stimulation (Fig. 1d), which was consistent with the mRNA results. These results suggest that MK2 does not control the expression of HuR.

### MK2 promotes cytoplasmic accumulation of HuR

**Fig. 2:**
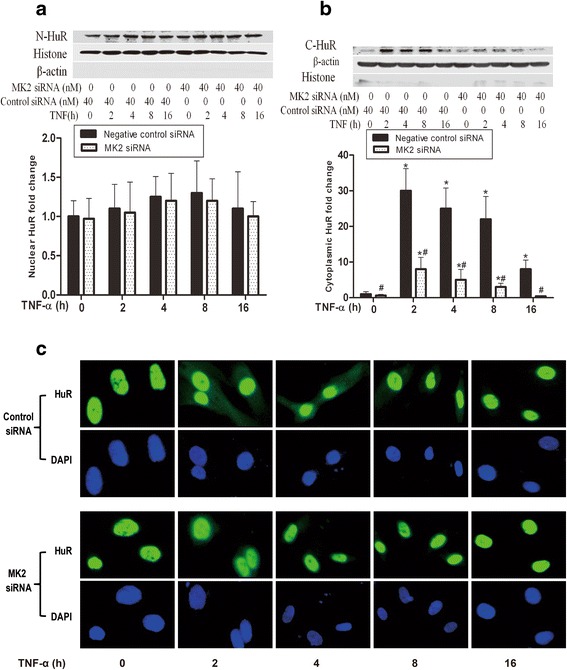
Knockdown of MK2 by inhibited TNF-α induced cytosolic accumulation of HuR. Lysates from control cells and MK2-silenced cells were subject to Western blot analysis for HuR, the densitometric analysis showed that MK2 silencing did not influence the nuclear HuR levels (**a**), however, reduced cytoplasmic accumulation of HuR compared to the control cells (**b**). The immunofluorescent staining for HuR (green) and DAPI (blue) showed that HuR was mainly accumulated in nucleus prior to TNF-α stimulation. Cytoplasmic HuR significantly increased after 2 h of TNF-α stimulation and then decreased after 8 h, while there was no remarkable change in nuclear HuR levels. Compared to the controls, MK2 silencing reduced cytoplasmic HuR but did not greatly affect the nuclear HuR levels (**c**). Western blot band density data are expressed as mean ± SEM. *n* =3, #*P* <0.05 vs. negative control siRNA-transfected cells, *P <0.05 vs. cells at TNF-α 0 h. Control siRNA, negative control siRNA N-HuR, nuclear HuR; C-HuR, cytoplasmic HuR

It has been observed that the active MK2 can induce cytoplasmic accumulation of HuR in HeLa cells [20]. Another study in MN-1 cells showed that p38 MAPK inhibition significantly reduced cytoplasmic accumulation of HuR [18]. Our previous and the present studies have shown that TNF-α stimulation can activate the p38 MAPK/MK2 cascade (Fig. 1b and reference 3). It is thus possible that activation of p38 MAPK/MK2 by TNF-α stimulation may promote the cytoplasmic accumulation of HuR in HPMECs. To this end, we analyzed the intracellular localization of HuR following MK2 silencing in the presence or absence of TNF-α. The nuclear HuR levels did not change significantly after TNF-α stimulation with or without MK2 knockdown (Fig. 2a). In sharp contrast, TNF-α stimulation significantly elevated the levels of cytoplasmic HuR, which were markedly reduced by MK2 siRNA (Fig. 2b). Further, the immunofluorescent staining for HuR (green) and DAPI (blue) showed that HuR was mainly accumulated in nucleus prior to TNF-α stimulation. Cytoplasmic HuR significantly increased after 2 h of TNF-α stimulation and then decreased after 8 h, while there was no remarkable change in nuclear HuR levels (Fig. 2c). Compared to the controls, MK2 siRNA reduced cytoplasmic HuR but had minimal effect on the nuclear HuR levels (Fig. 2c). These results suggest that MK2 promotes cytoplasmic accumulation of HuR after TNF-α stimulation in HPMECs.

### HuR silencing reduces TNF-α-induced expression of ICAM-1 but not IL-8 release

**Fig. 3:**
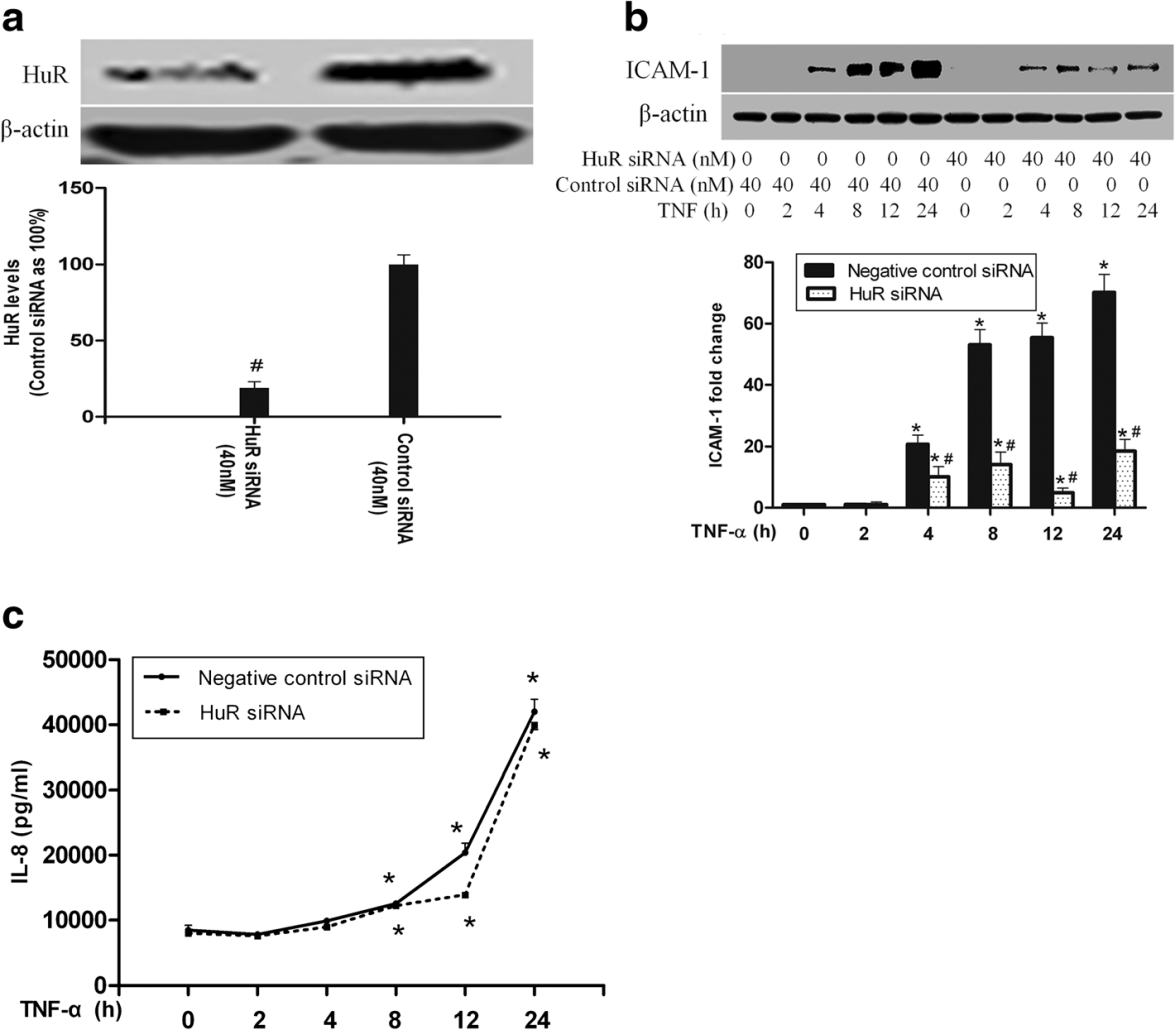
HuR silencing reduced ICAM-1 levels, while had no effect on IL-8 expression. The Western blot showed that HuR silencing greatly reduced HuR levels and down-regulated ICAM-1 levels following TNF-α stimulation (**a** and **b**). ELISA analysis demonstrated a significant increase of IL-8 both in HuR-silenced cells and control cells after TNF-α stimulation, nevertheless, the IL-8 levels in both groups were statistically undistinguishable (**c**). Western blot band density and ELISA data are expressed as mean ± SEM. n =3, #*P* <0.05 vs. negative control siRNA-transfected cells, *P <0.05 vs. cells at TNF-α 0 h. Control siRNA, negative control siRNA

It has been previously reported that HuR plays an important role in the post-transcriptional regulation of inflammatory factors expression such as vascular endothelial growth factor (VEGF) and IL-8 in malignant glioma cells and HCT8 cells, respectively [26, 27]. It is possible that the attenuation of cytoplasmic accumulation of HuR may contribute to the downregulation of ICAM-1 and IL-8, which was caused by TNF-α stimulation in MK2 silencing HPMECs. To test this hypothesis, we first examined the effect of HuR knockdown on TNF-α-induced ICAM-1 and IL-8 expression in HPMECs. HuR siRNA were transfected to cells for 24 h, and then cells recovered for another 24 h and stimulated with TNF-α. As shown in Fig. 3a, HuR siRNA (40 nM) significantly decreased HuR protein levels to 19 % of controls. TNF-α significantly induced the protein expression of both ICAM-1 (Fig. 3b) and IL-8 (Fig. 3c). HuR knockdown greatly reduced ICAM-1 levels compared to controls after TNF-α stimulation (Fig. 3b). In contrast to ICAM-1, the ELISA result showed HuR silencing had no effect on IL-8 release (Fig. 3c), which is not consistent with a previous study in HCT8 cells [26]. These results indicate the implication of HuR in the regulation of TNF-α-induced ICAM-1 expression without influencing IL-8 release in HPMECs.

### HuR increases the stability of ICAM-1 mRNA but not IL-8

**Fig. 4:**
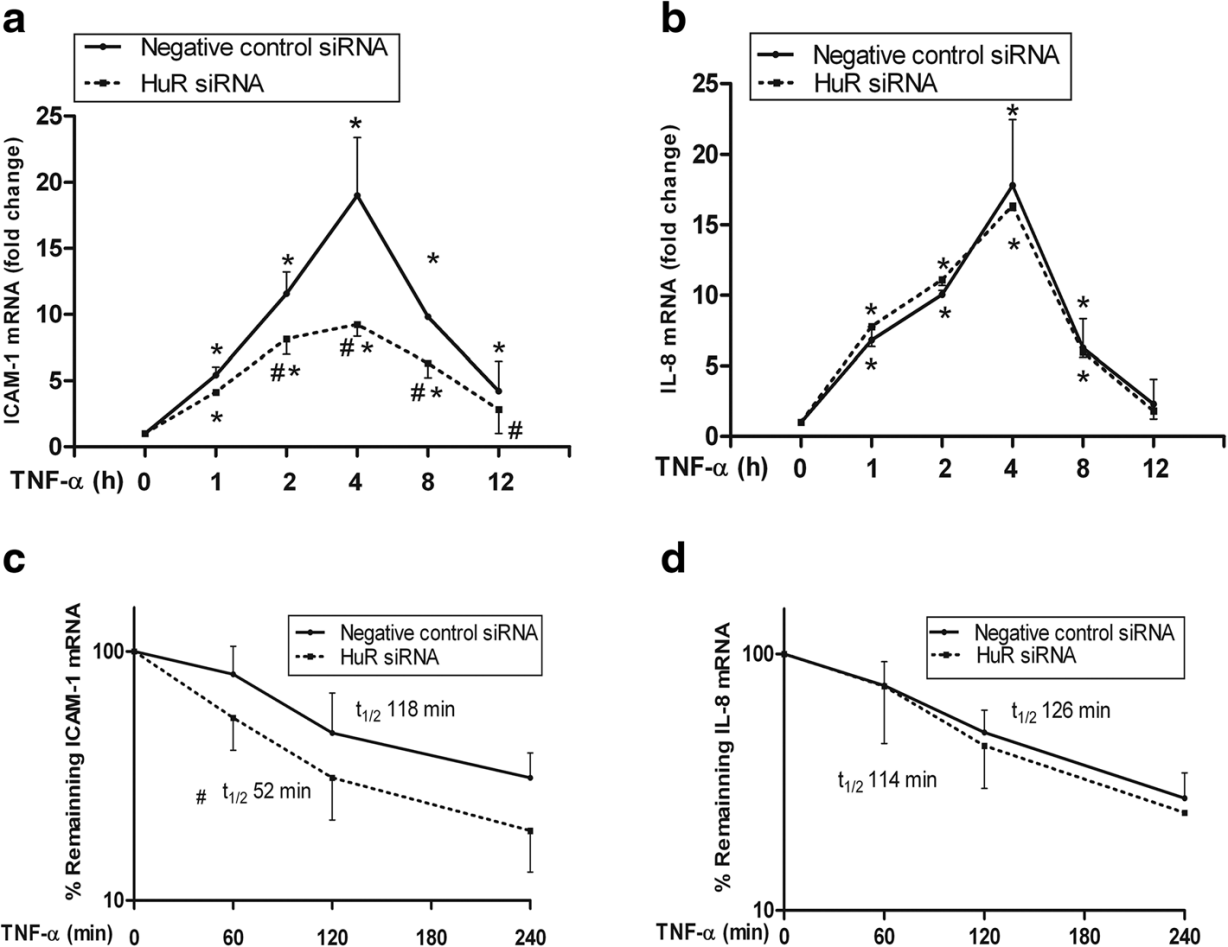
HuR affected the levels and stability of ICAM-1 mRNA without influencing IL-8 mRNA in HPMECs. Following TNF-α stimulation, the ICAM-1 and IL-8 mRNA levels were significantly increased in control cells, while HuR knockdown markedly reduced ICAM-1 mRNA levels but not IL-8 mRNA (**a** and **b**). HuR silencing decreased ICAM-1 mRNA stability, and the half-life was 118 min and 52 min in control cells and HuR-silenced cells, respectively (**c**). The half-life of IL-8 mRNA was 126 min and 114 min in control cells and HuR-silenced cells, respectively (**d**). Each point represents the mean ± SEM. n =3, #*P* <0.05 vs. negative control siRNA-transfected cells, *P <0.05 vs. cells at TNF-α 0 h

We next investigated whether HuR regulates the mRNA of ICAM-1 and IL-8. The real-time quantitative PCR results showed that ICAM-1 mRNA gradually increased and reached summit at 4 h of TNF-α stimulation, then went to baseline after 8 h in negative control siRNA-transfected cells. In sharp contrast, silencing of HuR significantly reduced ICAM-1 mRNA levels, and that was one fifth of controls 4 h after treatment with TNF-α (Fig. 4a). In control group, IL-8 mRNA increased to the top level at 4 h of TNF-α stimulation, while there was no significant difference of the IL-8 mRNA levels between control and HuR siRNA transfected cells (Fig. 4b). All datas demonstrated that HuR knockdown decreased ICAM-1 mRNA but does not influence IL-8 mRNA in HPMECs after TNF-α stimulation, which is consistent with the ICAM-1 and IL-8 proteins results (Fig. 3).

Subsequently, we explored whether HuR affects ICAM-1 and IL-8 mRNA stability. TNF-α stimulated HPMECs which were transfected with HuR or negative control siRNAs for 2 h, and then Act D (5 μg/ml) was added to stop gene transcription. After 0, 1, 2, and 4 h, total cellular RNA was harvested for real-time PCR to detect ICAM-1 and IL-8 mRNA levels. In control cells, the half-life of ICAM-1 mRNA after TNF-α stimulation was 118 min while was significantly reduced to 52 min in HuR-silenced HPMECs (*P* <0.05) (Fig. 4c). The half-life of IL-8 mRNA in control cells and in HuR-silenced HPMECs was comparable, which was 126 and 114 min, respectively (*P* >0.05). These results indicates a TNF-α-induced, HuR-dependent stabilization of ICAM-1 mRNA in HPMECs.

### HuR knockdown inhibits TNF-α-induced PMN adhesion to HPMECs

**Fig. 5:**
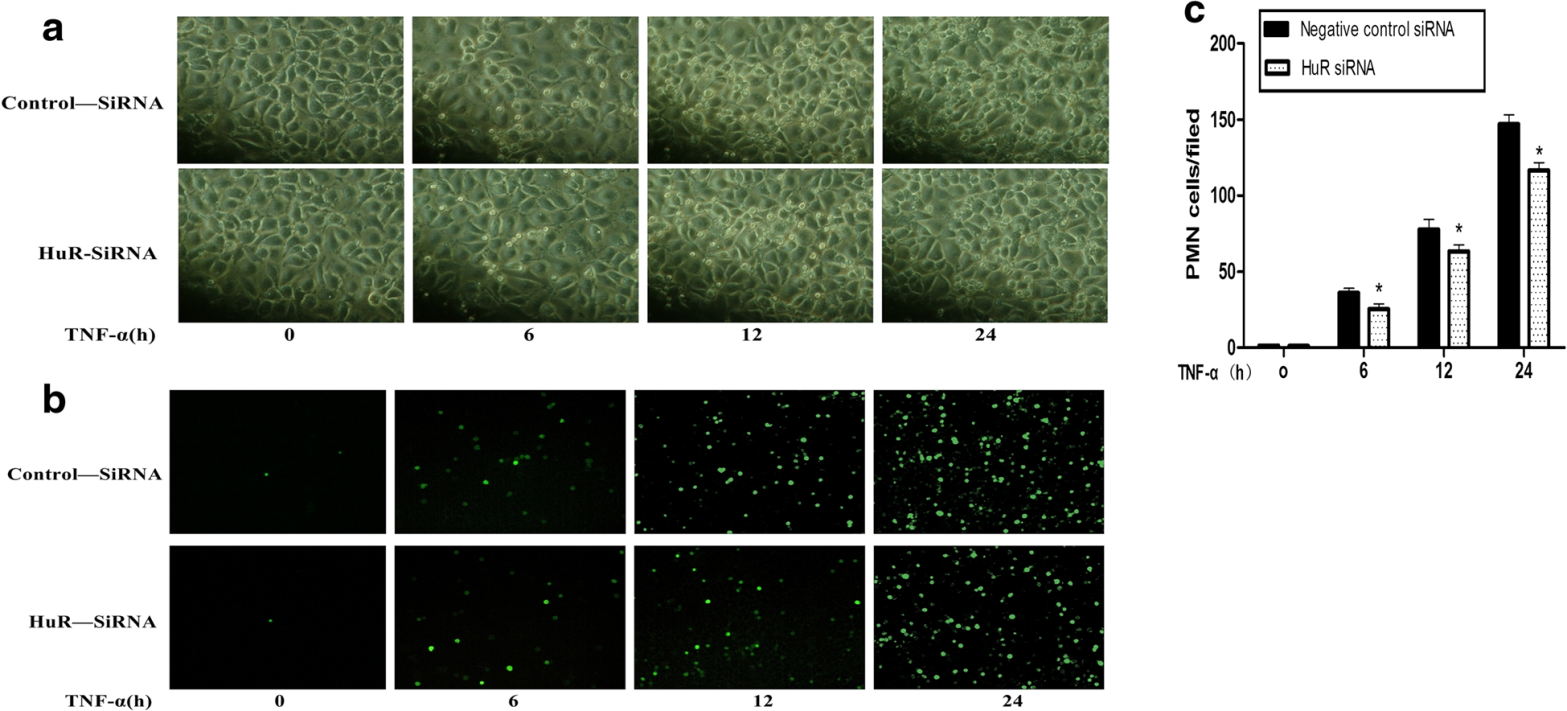
HuR knockdown inhibited PMN adhesion to TNF-α-induced HPMECs. HuR was silenced with HuR siRNA (40 nM), and then the HPMECs were stimulated with TNF-α (10 ng/ml) for 0 h, 6 h, 12 h and 24 h. BCECF/AM-labeled (green fluorescence) PMN were seed onto HUVEC and co-cultured for1 h. After removing the non-adherent cells, adherent cells were photographed and analyzed by microscopy. The pictures are representative optical fields (**a**) and fluorescence microscope (**b**) at different treatment (×200). (**c**) Quantitative analysis of the binding of PMN to HUVEC presented by bar graphs. Quantitative analysis are expressed as mean ± SEM. n =3, *P <0.05 vs. negative control siRNA-transfected cells. Control siRNA, negative control siRNA

ICAM-1 has been extensively studied from the standpoint of differentiating ALI from hydrostatic pulmonary edema and as the biomarker of prognosis of AIL/ARDS. Calfee et al. demonstrated that the ICAM-1 levels of plasma and pulmonary edema fluid are significantly higher in those with ALI/ARDS [28, 29]. ICAM-1 is found on the vascular endothelium and lung epithelium, and participates in transmigration of leukocytes to the site of inflammation [6, 30]. Our observation that HuR silencing reduced TNF-α-induced expression of ICMA-1, suggests that HuR may affect the interaction of neutrophil polymorphonuclear granulocytes (PMN) with HPMECs. For this, we examined the adhesion of PMN to TNF-α-activated HPMECs. Control confluent HPMECs incubated with PMN for 1 h showed minimal binding, but adhesion was gradually increased when the HPMECs were pretreated with TNF-a for 6 h, 12 h and 24 h (Fig. 5a and b). However, compared with TNF-a-treated HPMEC, silenceing of HuR with siRNA significantly reduced the number of PMN adhered to TNF-a-stimulated HPMEC (6 h, 12 h, 24 h) by 30 %, 18 % and 20 % inhibition, respectively (*P* <0.05) (Fig. 5b and c). These results suggest HuR promotes the adhesion of of PMN to TNF-α-activated HPMECs.

## Discussion

In this study, we found that knockdown of MK2 by siRNA did not affect the expression but significantly impaired the cytoplasmic accumulation of HuR after TNF-α stimulation in HPMECs. We further revealed that HuR knockdown decreased the mRNA and protein levels of ICAM-1 in HPMECs after TNF-α stimulation. Most importantly, silencing of HuR resulted in destabilization of the mRNA of ICAM-1 and inhibition of TNF-α-induced PMN adhesion to HPMECs. Our results suggest that MK2 regulates TNF-α-induced ICAM-1 expression by altering the cytoplasmic localization of HuR in HPMECs.

HuR is a member of the embryonic lethal abnormal visual family and can bind to the ARE motif such as AUUUA in the 3′-UTR of mRNAs. The cytoplasmic localization of HuR increases its binding to the AREs and stabilizes the ARE-containing mRNAs [26, 27, 31, 32]. Our previous studies have shown that MK2 up-regulates ICAM-1 and IL-8 expression following TNF-α stimulation in HPMECs. However, the downstream protein by which MK2 regulates ICAM-1 and IL-8 expression is unknown. A previous study has shown that MK2 can promote cytoplasmic accumulation of HuR in HeLa cells [20]. To investigate whether MK2/HuR pathway is involved in the regulation of ICAM-1 and IL-8 expression in HPMECs, we firstly examined the effect of MK2 on HuR expression. Our study indicates that HuR mRNA and protein level changed little after TNF-α activation in presence or absence of MK2 silencing neither, implying that MK2 is not involved in the regulation of HuR expression. Further, we found that HuR was mainly located in nucleus, and there was very little HuR in cytoplasm before TNF-α activation. TNF-α activation greatly increased MK2-dependent cytoplasmic accumulation of HuR. These results indicate that the subcellular localization of HuR is regulated by MK2 in TNF-α-activated HPMECs. It should be noted that the levels of nuclear HuR did not decrease concomitantly with the increase of cytoplasmic HuR. A possible explanation is that there is so little HuR in cytoplasm that small parts of HuR translocated to cytoplasm leads to a significant increase in cytoplasmic HuR but not detectable alteration of HuR in nuclei.

**Fig. 6:**
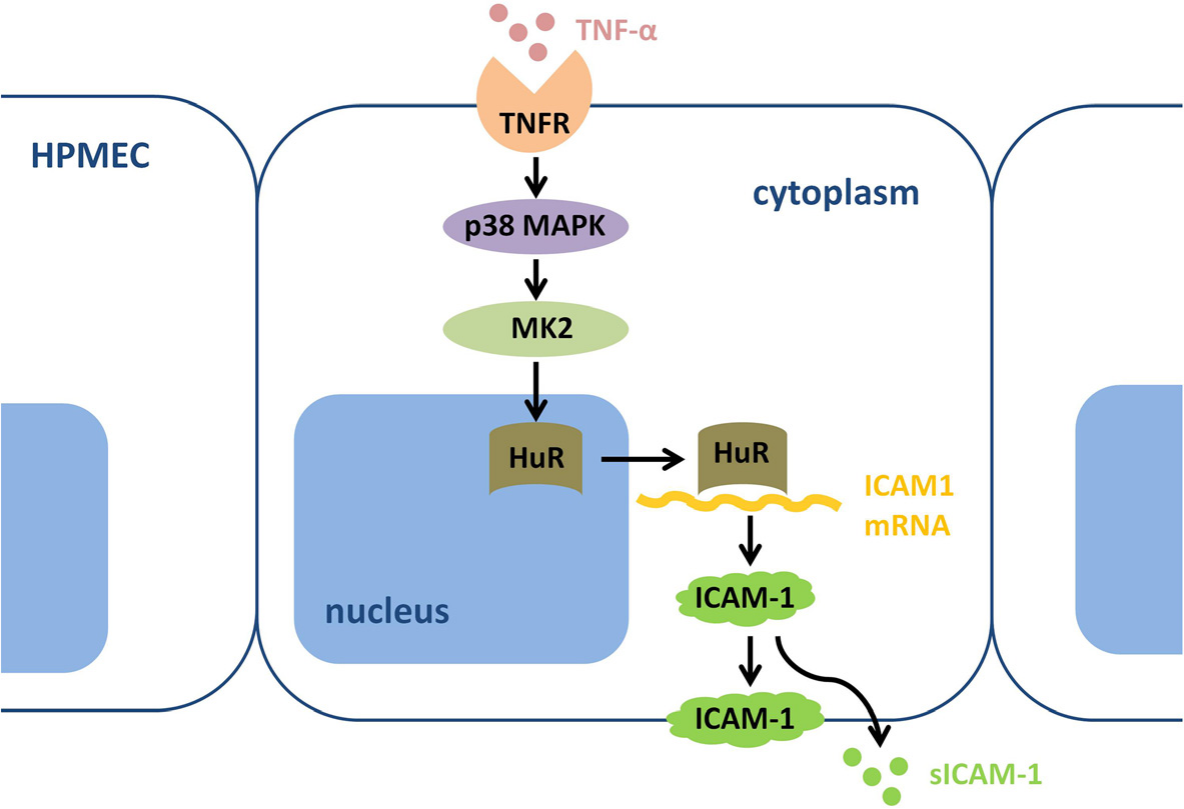
Schematic model of the MK2/HuR pathway in the post-transcriptional regulation of TNF-α-induced ICAM-1 expression. The present results show that HuR nucleo-cytoplasmic shuttling mediated by TNF-α stimulation results in the stabilization of ICAM-1 mRNAs and increased protein production. The possible mechanism is that MK2 induces HuR cytoplasmic translocalization, which leads to an increase in stability of HuR-mRNA complex formation, and therefore increases ICAM-1 expression. TNFR, TNF-receptor; sICAM-1, soluble ICAM-1

HuR has been reported to regulate the expression of several factors such as VEGF, IL-8 and survivin by stabilizing these ARE-bearing mRNAs in other cells [14, 26, 27, 33]. Similar to those results, our present study showed that HuR silencing reduced the mRNA stability and protein levels of ICAM-1. However, in contrast to the previous two studies in malignant glioma cells and HCT8 cells [26, 27], HuR had no effect on IL-8 expression in TNF-α-stimulated HPMECs. Considering malignant glioma cells and HCT8 cells are both cancer cells, we suppose that some factor(s) present in HPMECs but absent in cancer cells may compensate for the lack of HuR in regulating IL-8 but not ICAM-1 after TNF-α stimulation. We further found that the ICAM-1 mRNA in HuR-silenced cells became less stable and the half-life was remarkably decreased compared to that in control groups. Nevertheless, the IL-8 mRNA levels and stability in control cells and that in HuR-silenced cells were similar. Taken our previous studies together, we conclude for the first time that the MK2/HuR pathway is involved in the regulation of TNF-α-induced ICAM-1 expression by altering the mRNA stability (Fig. 6).

To date, the exact mechanisms of ALI/ARDS are not completely known. The present results demonstrated that knockdown of HuR inhibited neutrophil adherence to the HPMECs. Our findings suggest that the MK2/HuR pathway may play an important role in acute inflammatory response in HPMECs and may become an effective treatment target for ALI/ARDS in future.

Although the latest research indicates that the MK2/HuR pathway regulated ICAM-1 expression after TNF-α stimulation in HPMECs, the mechanisms by which MK2 regulates HuR translocation are not completely understood. Doller et al. [34] have reviewed several signaling pathways regulating nucleo-cytoplasmic shuttling of HuR, and pointed out that the protein sequence of HuR has no putative phosphorylation sites for MAPKs and HuR may probably not be phosphorylated by MK2. However, a study in RKO cells showed that HuR is phosphorylated at Thr118 by p38 MAPK and the Thr118 phosphorylation contributes to cytosolic localization of HuR induced by γ radiation [35]. Further studies are needed to clarify whether MK2 promotes cytoplasmic localization of HuR by phosphorylating Thr118 of HuR in TNF-α-stimulated HPMECs. It should be noted that, in addition to HuR, there are many other ARE-binding proteins such as tristetraprolin [36] and hnRNP A0 [37], it will be of importance to assess whether other ARE-binding proteins are involved in the regulation of ICAM-1 and IL-8 expression and whether other ARE-binding proteins interact with HuR.

## Conclusions

In summary, our results showed that the MK2/HuR signaling axis regulated TNF-α-induced ICAM-1 expression by promoting the stabilization of ICAM-1 mRNA in HPMECs, and silencing of HuR impaired TNF-α-induced adhesion of neutrophils to HPMECs. Our findings suggest that the MK2/HuR pathway play an important role in acute inflammatory response in HPMECs and targeting MK2/HuR signaling is a promising strategy for the development of an effective treatment for ALI/ARDS.

## Abbreviations

ALI: acute lung injury
ARDS: acute respiratory distress syndrome
ICAM-1: intercellular adhesion molecule-1
IL-8: interleukin-8
MAPK: mitogen-activated protein kinase
MK2: mitogen-activated protein kinase p38/activated protein kinase 2
HPMECs: human lung microvascular endothelial cells
TNF-α: tumor necrosis factor-α
HuR: human antigen R
AREs: AU-rich elements
ActD: actinomycin D
TLR4: toll-like receptor 4
SMN: survival motor neuron
VSMCs: vascular smooth muscle cells
uPA: urokinase plasminogen activator
PMN: polymorphonuclear granulocytes
VEGF: vascular endothelial growth factor
